# Compact slow-light waveguide and modulator on thin-film lithium niobate platform

**DOI:** 10.1515/nanoph-2023-0306

**Published:** 2023-08-23

**Authors:** Gengxin Chen, Haohua Wang, Bin Chen, Ziliang Ruan, Changjian Guo, Kaixuan Chen, Liu Liu

**Affiliations:** State Key Laboratory for Modern Optical Instrumentation, College of Optical Science and Engineering, International Research Center forAdvanced Photonics, Zhejiang University, Hangzhou 310058, China; Guangdong Provincial Key Laboratory of Optical Information Materials and Technology, South China Academy of Advanced Optoelectronics, Sci, Bldg. No.5, South China Normal University, Higher-Education Mega-Center, Guangzhou 510006, China; National Center for International Research on Green Optoelectronics, South China Normal University, Guangzhou 510006, China; Jiaxing Key Laboratory of Photonic Sensing & Intelligent Imaging, Intelligent Optics & Photonics Research Center, Jiaxing Research Institute, Zhejiang University, Jiaxing 314000, China

**Keywords:** thin-film lithium niobate, Bragg grating, electro-optic modulator, slow-light effect

## Abstract

Lithium niobate Mach–Zehnder modulators (MZMs) with compact footprint and fast electro-optics (EO) responses are highly demanded for the next-generation optical interconnect systems. Here, we demonstrate slow-light (SL) effect using a coupled Bragg resonator structure on the thin-film lithium niobate (TFLN) platform, and an ultra-compact SL-MZM with length *L* of ∼370 μm is also constructed. The fabricated SL waveguides show a large optical passband width of ∼8 nm, an insertion loss of 2.9 dB, and a maximal optical group index of 7.50, corresponding to 3.4 times as large as that of regular TFLN rib waveguide. The fabricated SL-MZM exhibits a large EO bandwidth of >50 GHz in an operating wavelength band of ∼8 nm as well. High-speed OOK transmissions at data rates of 64 Gbit/s and 80 Gbit/s are successfully achieved. To our best knowledge, it is first time to build SL waveguides and compact SL-MZMs with large EO bandwidths of >50 GHz on the monolithic TFLN platform.

## 1 Introduction

Thin-film lithium niobate (TFLN) has emerged as a versatile platform for low-loss integrated photonics with applications in optical communications [1], nonlinear optics [2], and quantum optics [3]. Recently, high-performance TFLN-based electro-optic (EO) modulators from visible [4] to near-infrared [5, 6] band have been demonstrated with potential applications in the next generation of high-speed optical interconnect systems. However, due to the moderate EO coefficient (*r*_33_ ∼ 27 pm/V) of the lithium niobate material, traditional Mach–Zehnder modulators (MZMs) [7, 8] on TFLN feature lengths of >1 cm. The device footprint can be reduced by using cavity structures, such as micro-ring (MR) resonator [9], Fabry–Perot (FP) cavity [10, 11], and photonic-crystal (PC) waveguides [12]. Although these cavity-based modulators possess a much more compact size, their operating wavelengths are limited in a narrow passband (<1 nm), which then requires dedicated wavelength-tuning mechanisms. In addition, there is normally an intrinsic trade-off lies between driving voltage and modulation speed in cavity-based modulators [13]. A high quality factor (Q-factor) resonator can effectively reduce the driving voltage, while decreases the modulation speed significantly due to the longer photon lifetime inside the resonator, which is not desired for high-speed data transmissions. Another strategy to achieve compact EO modulators is based on plasmonic effect [14], which facilitates to obtain the highest modulation efficiency of ∼0.23 V cm so far on TFLN with a compact footprint. Although a high modulation efficiency can be achieved, such a device suffers from a great optical loss of ∼20 dB. Secondly, the half-wave voltage of ∼150 V is also ultra-high so that swinging such a large voltage at high speed is difficult for any driver circuits. Moreover, due to the poor high-frequency performance, the measured EO response is limited to 10 GHz. The above issues make the plasmonic modulators difficult to use practically. Therefore, a novel design of compact EO modulators is still expected on the TFLN platform.

Slow-light (SL) effect is a fundamental physical phenomenon, which can reduce the group velocity of light using, e.g., specific engineered structures on photonic integrated circuits. The SL effect can also increase the interaction between the light and the electric field in an optical modulator, which then helps improve the modulation efficiency. SL modulators based on PC waveguides have been experimentally demonstrated on silicon-on-insulator (SOI) platform [15]. A large optical bandwidth over 10 nm with a large optical group index (*n*_g_) of 34 can be achieved [16]. However, PC waveguides still suffer from a small fabrication tolerance and, hence, require a high-precision manufacturing process. The Bragg grating waveguides, another periodic waveguide structure, possess much simpler design strategies of SL structures than that of PC waveguides. SL-MZMs [17] assisted by coupled waveguide Bragg resonators can achieve a compact footprint of hundreds of microns with an improved modulation efficiency and sub-fj/bit power consumption, as well as a better stability over a large temperature range. More recently, a compact ultra-high bandwidth over 110 GHz silicon-based SL-MZM with on-off keying (OOK) data transmission beyond 110 Gbit/s has been demonstrated successfully [18]. Although the SL effect has been verified in silicon-based modulators, there are few demonstrations on the TFLN platform, and the SL effect on it is still far from fully exploited. On the TFLN platform, periodic dielectric waveguide structure [19] utilizing SL effects has been adopted to achieve high-speed modulation, but the full-etched TFLN waveguide is difficult to achieve a low-loss propagation and the calculated optical *n*_g_ is relatively small (∼2.665), which also lacks of an experimental verification. Although using Bragg gratings [20] can help achieve a high modulation efficiency in SL-MZMs on the hybrid silicon nitride/TFLN platform, the SL effect only appears at the edge of the passband in this case, which induces a large propagation loss, and the operating wavelength band is also limited below 1 nm.

In this paper, we theoretically and experimentally demonstrate SL waveguides using a coupled Bragg resonator structure. The optical transmissions of SL waveguides show obvious and flat passbands, and the largest optical *n*_g_ of 7.50 can be achieved, corresponding to 3.4 times as large as that of a regular TFLN rib waveguide. Moreover, this SL waveguide, at the measured maximal optical *n*_g_, exhibits a large optical passband width BW_3dB_ of ∼8 nm, as well as a low insertion loss (IL) of ∼2.9 dB, which is better than traditional Bragg grating structures. In order to further exploit this SL effect, compact traveling-wave SL-MZMs are also fabricated, showing a large EO modulation bandwidth of >50 GHz in a wavelength range from 1557.43 nm to 1565.24 nm. The high-speed OOK modulation at data rates of 64 Gbit/s and 80 Gbit/s are also successfully achieved with a dynamic ER of ∼2 dB. To our best knowledge, it is the first time to demonstrate the SL effect and SL-MZMs on monolithic TFLN platform.

## 2 Simulation and device design

**Figure 1:**
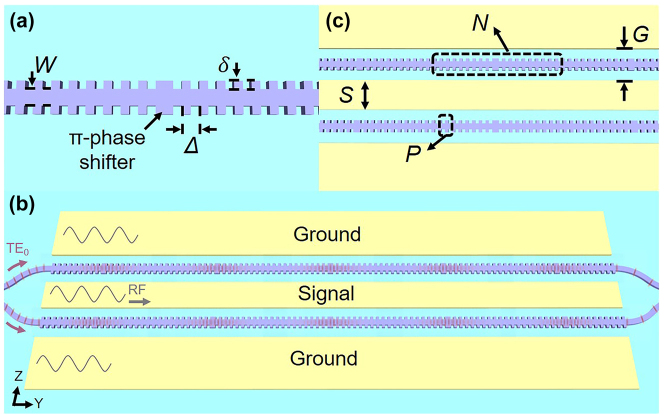
Schematic configuration of the proposed SL waveguide and modulator. (a) Top view of the coupled Bragg resonator based SL waveguide that consists of series of fishbone-like Bragg gratings separated by a *π*-phase shifter region. (b) Schematic view of the proposed traveling-wave SL-MZM. (c) Top view of the modulation region including multiple cascaded–coupled Bragg resonators.

A detailed schemetic drawing of the proposed SL waveguide is shown in Figure 1(a), which consists of series of fishbone-like Bragg gratings with period Δ separated by a half-period *π*-phase shifter. A waveguide resonator unit is, therefore, formed. The fishbone-like Bragg gratings are designed using sidewall corrugations of a width *δ* and a duty cycle 50 % on a halfly etched TFLN waveguide of a width *W* and a total thickness of 400 nm. The period Δ is set to 461 nm to ensure that the Bragg reflection wavelength is centered around 1550 nm. In order to demonstrate the SL effect inside EO modulators, the proposed SL-MZM is also shown in Figure 1(b). The SL waveguide is arranged on both arms of the SL-MZM, and the ground-signal-ground (GSG) traveling-wave electrode works in a push–pull configuration. The radio-frequency (RF) signal, as well as the fundamental transverse-electrical (TE_0_) polarized optical mode, transmits along the *y*-direction of the *x*-cut TFLN chip. The *z* axis of the lithium niobate material is perpendicular to the waveguide, so that the highest EO coefficient could be employed for modulation. As it can be seen in Figure 1(c), each arm of SL-MZM consists of cascaded Bragg resonators of total number *N*, and adjacent resonators are separated with Bragg grating mirrors of total number of period *P*.

**Figure 2:**
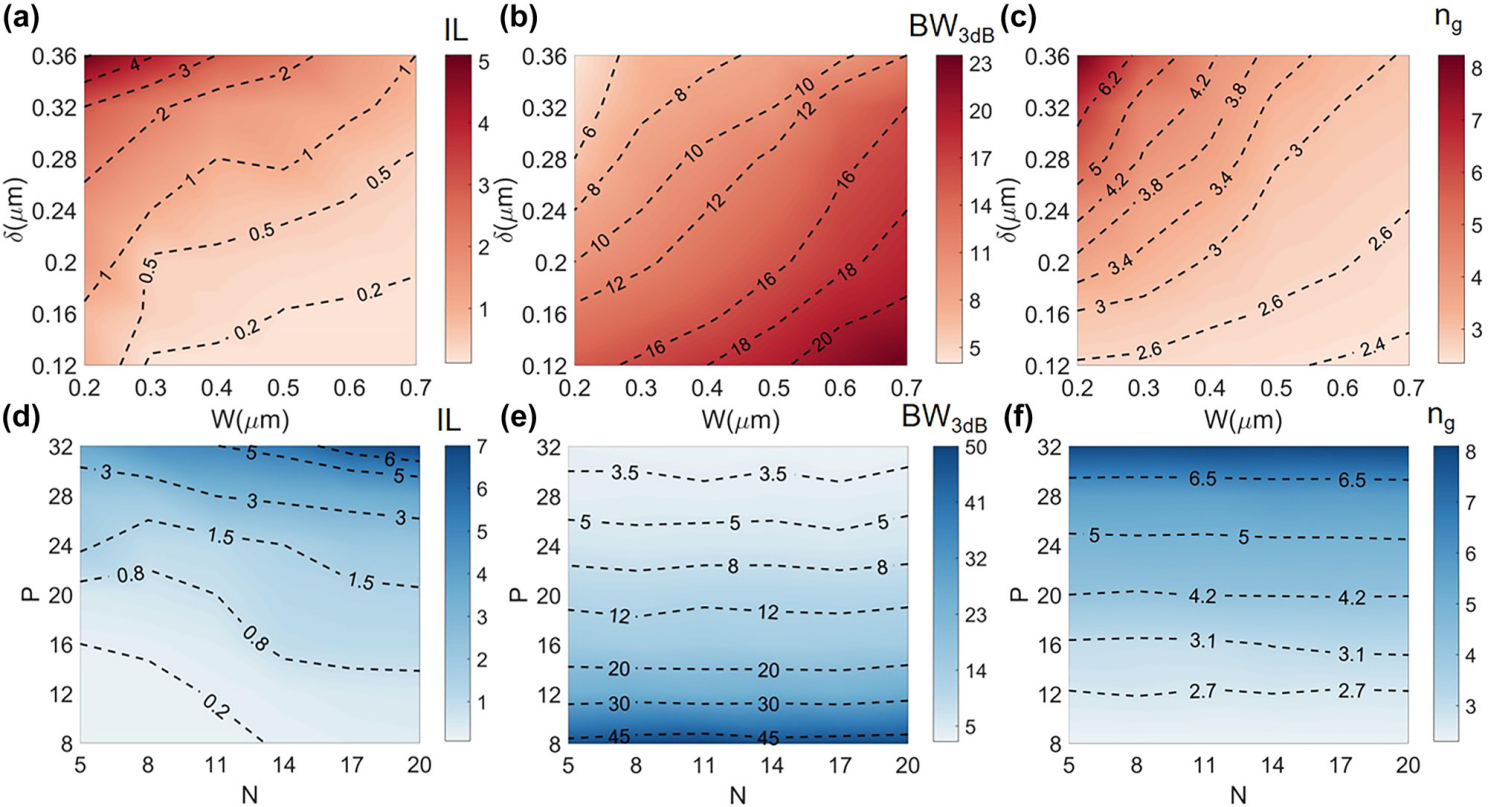
Pseudo-color plots of some simulated SL performance figures of (a) & (d) IL, (b) & (e) optical passband width BW_3dB_, and (c) & (f) optical group index *n*_g_ with different structural parameters. The pseudo-color maps in red are related to *δ* and *W*, with fixed *P* = 20, *N* = 5. The pseudo-color maps in blue are related to *P* and *N*, with fixed *W* = 0.4 μm, *δ* = 0.24 μm.

In order to understand the SL effect of the proposed cascaded Bragg resonators on a TFLN waveguide, we firstly investigate the mode characteristics of the overall structure, including IL, optical passband width BW_3dB_ (defined as the wavelength range where the transmission drop 3 dB from its peak value within the passband), and optical group index *n*_g_. The performances of these figures under different structural parameters, i.e., *N*, *P*, *W*, and *δ*, have been shown in Figure 2. Clearly, the decreased waveguide width *W* and increased corrugation depth *δ* of the Bragg grating can effectively enhance SL effect but at the expense of increased IL and decreased BW_3dB_. At the same time, the increased number of Bragg grating period *P* between each cavity also has a similar impact. These trends can be explained as the Bragg reflection becomes stronger when the relative corrugation and length of the grating increases. The coupling between adjacent resonators is, therefore, decreased, leading to a slow propagation of the light along the whole cascaded structure, i.e., the SL effect. Intuitively, the increased group index will also result in a higher propagation loss, and the weakened coupling between resonators will decrease the optical passband as well. On the other hand, the total number *N* of resonators forming the waveguide does not affect the SL effect but the overall IL of the device, since it simply becomes longer, as shown in Figure 2(d).

**Figure 3:**
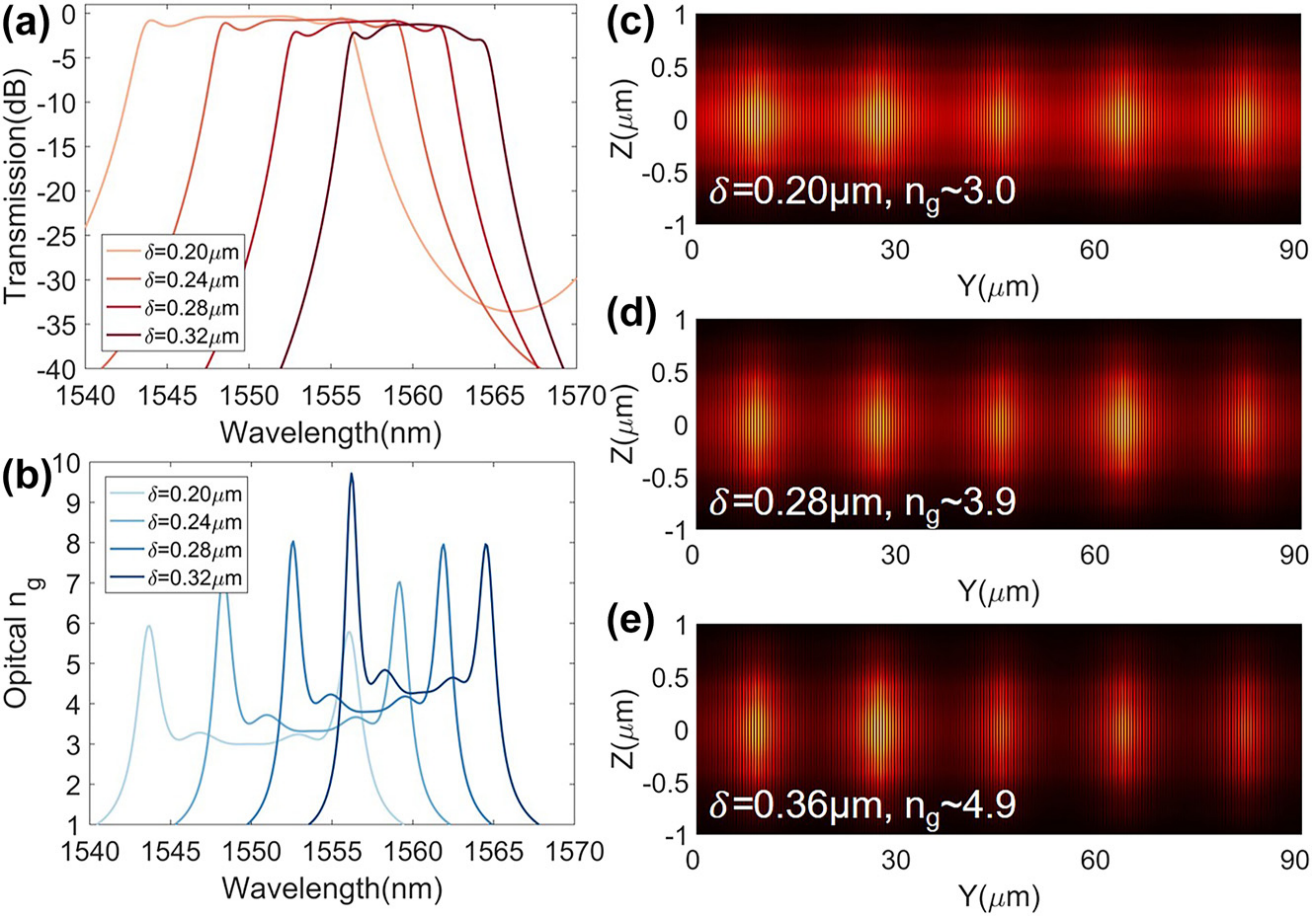
Simulated (a) optical transmission spectra and (b) the corresponding optical group index *n*_g_ at different wavelengths for different *δ*. Simulated electric field intensity distributions inside the SL waveguides for (c) *δ* = 0.20 μm (*n*_g_ = ∼3.0), (d) *δ* = 0.28 μm (*n*_g_ = ∼3.9), and (e) *δ* = 0.36 μm (*n*_g_ = ∼4.9). Here, *W* = 0.4 μm, *P* = 20, and *N* = 5.

Next, we further simulate optical transmission spectra and the corresponding wavelength response curves of the optical group index *n*_g_ with different corrugation depth *δ* in the wavelength range of 1.5 μm–1.6 μm as shown in Figure 3(a) and (b). A clear passband with flat transmission and *n*_g_ curves can be obtained, which indicates the robustness of the present coupled resonator SL structure as compared to the Bragg gratings [20]. When the corrugation width *δ* increases, the simulated transmission spectra appear a significant red-shift. Moreover, the passband width BW_3dB_ becomes narrower, and the IL of the SL waveguides increases slightly. These are all consistent with the former analyses. Figure 3(c) and (d) show the simulated electric field intensity distributions inside SL waveguides with *n*_g_ = ∼3.0, *n*_g_ = ∼3.9, and *n*_g_ = ∼4.9, respectively. Clear coupling between adjacent resonators and the enhancement of SL effect with increased *δ* can be observed as the field energy concentrates more inside the resonators. Therefore, from above analyses, by carefully designing length and grating parameters of the proposed structure, we can obtain efficient SL waveguides with a tuned optical *n*_g_ and BW_3dB_.

**Figure 4:**
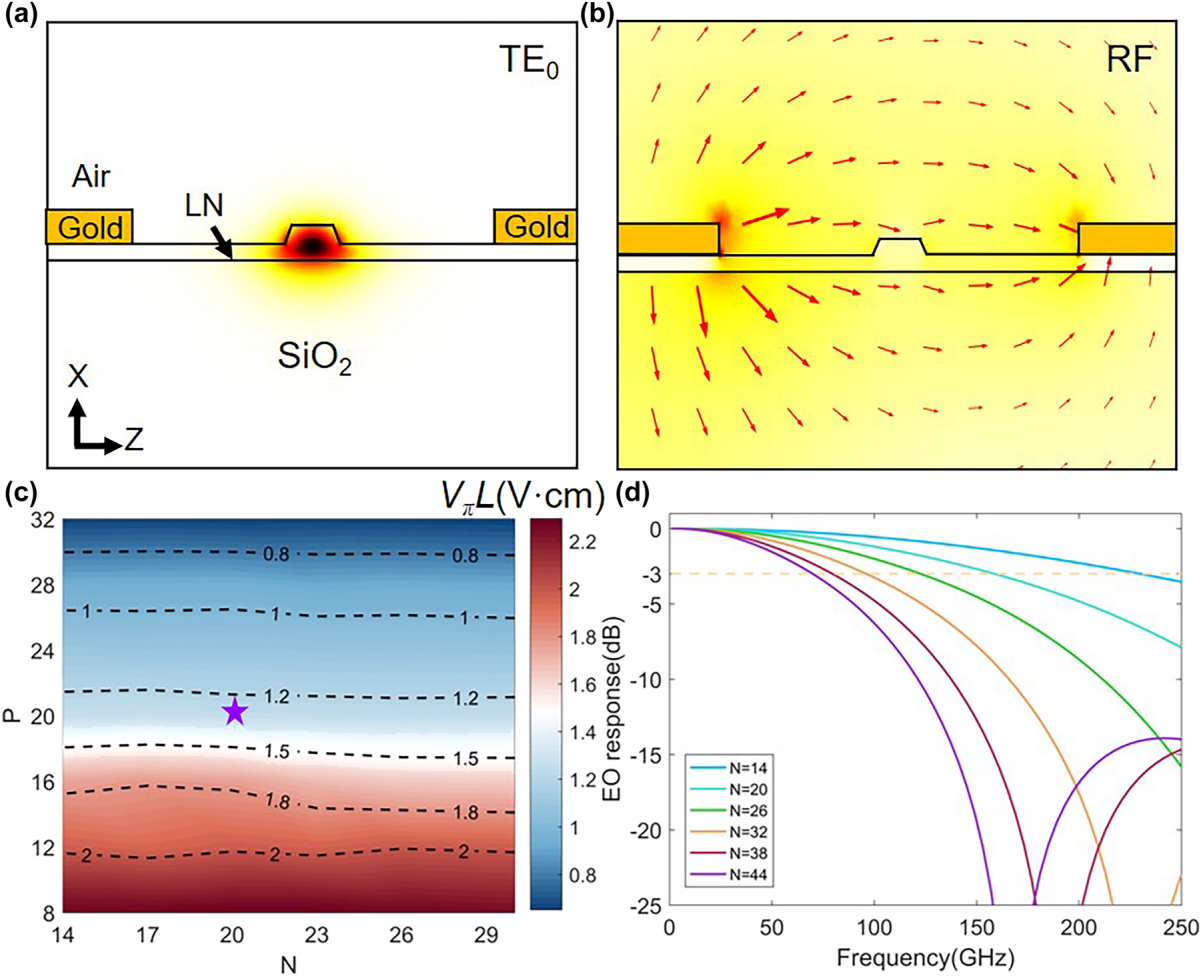
Design of SL modulator. (a) Simulated electric field distribution of the fundamental TE_0_ optical mode at a wavelength of 1.55 μm in the TFLN waveguide. (b) Simulated microwave field distribution at 100 GHz. (c) Pseudo-color plots for the modulation efficiency *V*_*π*_*L* with different *N* and *P*. Here, *W* = 0.4 μm, *δ* = 0.24 μm. (d) Normalized EO responses with different *N*, corresponding to different lengths of the modulation section. The star in (c) marks the optimal design adopted in fabrication.

To further investigate the EO interaction between the SL waveguide and the modulation electrode, the numerically simulated electric field of the fundamental TE_0_ optical mode at the wavelength of 1.55 μm is plotted in the cross section of the waveguide as shown in Figure 4(a). The electrode gap *G* and signal electrode width *S* is set as 5.3 μm and 16 μm, respectively, to ensure a perfect impedance matching and an efficient modulation with a low metal absorption loss <0.01 dB/cm. Figure 4(b) shows the simulated microwave field distribution at frequency of 100 GHz. To examine the modulation efficiency, a key performance figure, i.e., half-wave voltage length product (*V*_*π*_*L*), is defined and simulated. As shown in Figure 4(c), increasing *P* can largely improve the modulation efficiency due to the enhancement of SL effect as discussed above. On the other hand, increasing the number *N* of the cascaded resonators would not affect the modulation efficiency. Yet, due to the longer modulation length in this case, the modulation depth or the drive voltage performances can be improved. However, due to the SL effect, a large velocity mismatch between the microwave signal (with an index of 2.18) and the optical wave exists. The longer modulation length will aggregate the walk-off effect between them, and hence, the EO modulation damps quickly at high frequencies as shown in Figure 4(d). Therefore, a modulation length *L* of ∼370 μm with *N* = 20, *P* = 20 is adopted for the fabrication finally to give a reasonable modulation efficiency of ∼1.25 V cm and a high EO bandwidth of ∼160 GHz, which is marked as a red star in Figure 4(c). The structural parameter range was selected for achieving optimal performances for SL waveguides and modulators centered at 1550 nm.

## 3 Fabrication and measurement

**Figure 5:**
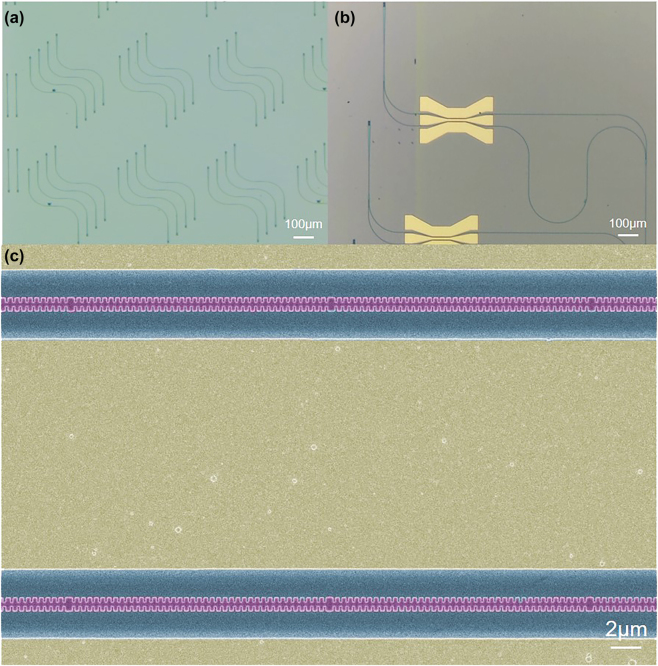
Optical microscope images of fabricated (a) SL waveguides and (b) SL-MZMs. (c) Scanning electron microscopy picture of the modulation section of a fabricated SL-MZM.

Figure 5(a) and (b) show a series of fabricated SL waveguides and SL-MZMs. Grating couplers were also prepared [21, 22] for light in and out coupling for measurement purposes. These devices were fabricated on a commercial *x*-cut lithium-niobate-on-insulator wafer (NanoLN) with a 400-nm-thick top lithium niobate layer and a 3-μm-thick buried oxide layer on a Si substrate. First, the TFLN waveguide was patterned using an electron beam lithography (EBL) system (Raith VOYAGER) with a 300-nm-thick negative photoresist. Subsequently, the mask pattern was transferred to the TFLN layer with 200 nm lithium niobate etched using Ar plasma. Then, metal electrode pattern was then created using ultra-violet contact lithography, and the metal electrode layer composed of 5 nm Ti and 400 nm Au was deposited using electron-beam evaporation and lift-off processes.

**Figure 6:**
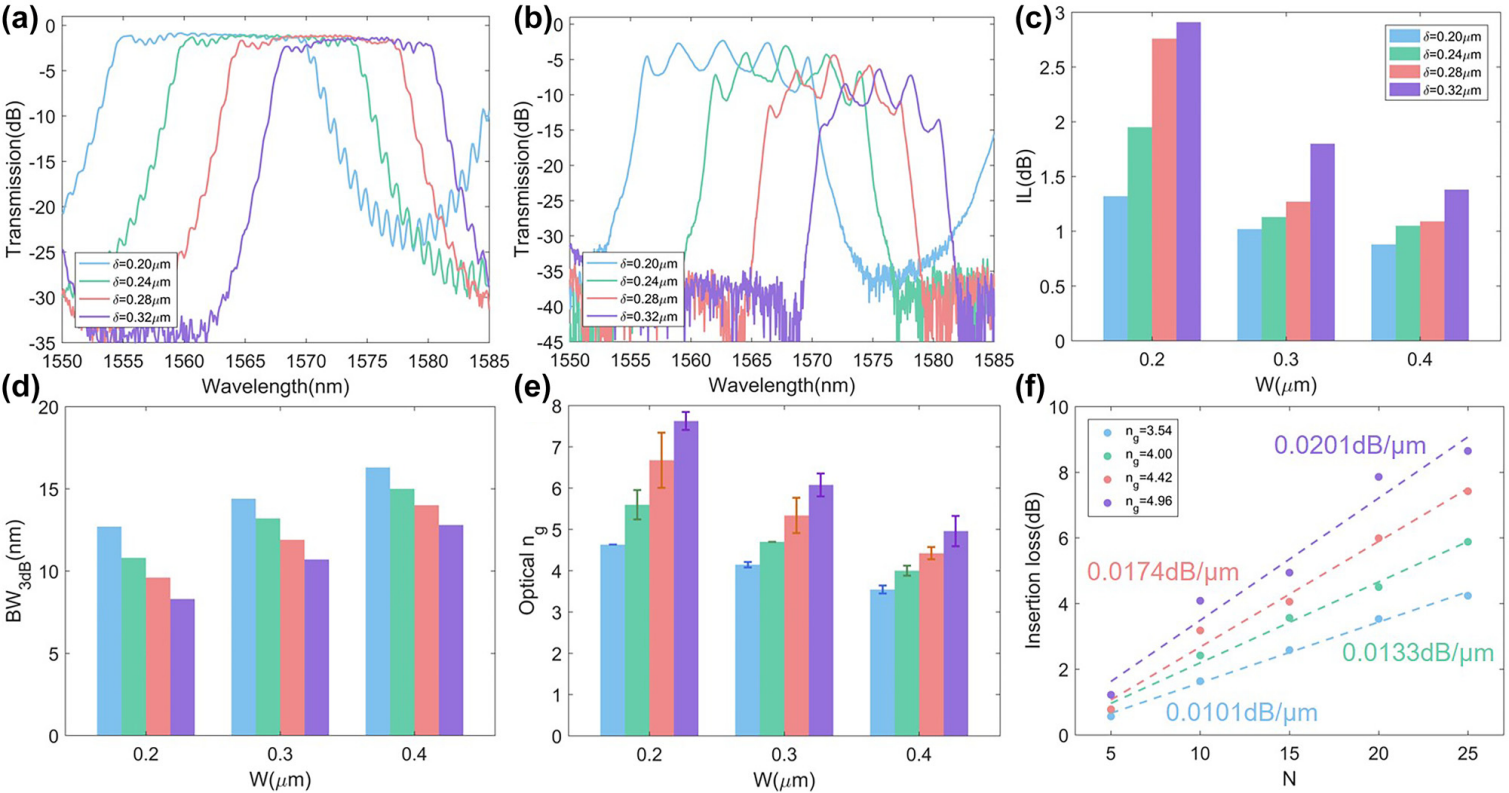
Measured optical transmissions for SL waveguides (a) without FP cavities and (b) with additional FP cavities for different *δ* with *W* = 0.4 μm, *P* = 20, and *N* = 5. The measured (c) IL, (d) BW_3dB_, and (e) optical *n*_g_ extracted from the structures with FP cavities for different *δ* and *W*. The error bars show the fluctuation of extracted optical *n*_g_. (f) Measured optical IL for different optical *n*_g_ as a function of *N* with *W* = 0.4 μm and *P* = 20. The optical ILs per unit length are also marked in the corresponding colors.

First, a series of SL waveguides with different structural parameters are characterized. The optical transmissions exhibit clear passbands as shown in Figure 6(a). Except that the central wavelengths of transmission spectra exhibit a slight red-shift, the experimental results here are in good agreement with the simulations in Figure 3(a). In order to quantitively characterize their *n*_g_, additional FP cavity structures were also fabricated, which consists of not only the SL waveguides themselves but also more Bragg gratings at the input and output sides of the SL waveguides. The extra number of gratings is set to 20. These extra Bragg gratings would decrease the coupling of the first and last resonator units to the input and output waveguides, respectively. Effectively, this helps construct an FP cavity with the SL waveguide embed. As shown in Figure 6(b), the fabricated FP cavities show obvious dips in the transmission spectra, which is resulted from the FP resonances. They can be used to derive the optical group index *n*_g_ of the corresponding embed SL waveguides as *n*_g_ = *λ*^2^/(2 × *F* × *L*), where *λ* is the central wavelength of the SL waveguides, *F* is free spectrum range of transmission dips, and *L* is the physical length of SL waveguide in the cavity. It is clearly seen from Figure 6(c)–(e) that the variation trends of IL, BW_3dB_, and optical *n*_g_ are well matched to our simulated results discussed in Section 2. The SL waveguide with the maximal derived optical *n*_g_ of ∼7.5 shows a low IL of ∼2.9 dB and a large BW_3dB_ of ∼8 nm, whose derived optical *n*_g_ is nearly 3.4 times as large as that of a TFLN rib waveguide (*n*_g_ ∼ 2.2) [7]. This large optical passband width enables a large fabrication tolerance for variations in, e.g., the waveguide width, the total LN thickness, and the etched depth. In order to further investigate the propagation loss of the SL waveguides, the measured insertion losses at different optical *n*_g_ as a function of waveguide lengths (i.e., different *N*) are shown in Figure 6(f). The propagation losses per unit length of 0.0101 dB/μm, 0.0133 dB/μm, 0.0174 dB/μm, and 0.0201 dB/μm are fitted for SL waveguides with *n*_g_ = 3.54, *n*_g_ = 4.00, *n*_g_ = 4.42, and *n*_g_ = 4.96, respectively.

**Figure 7:**
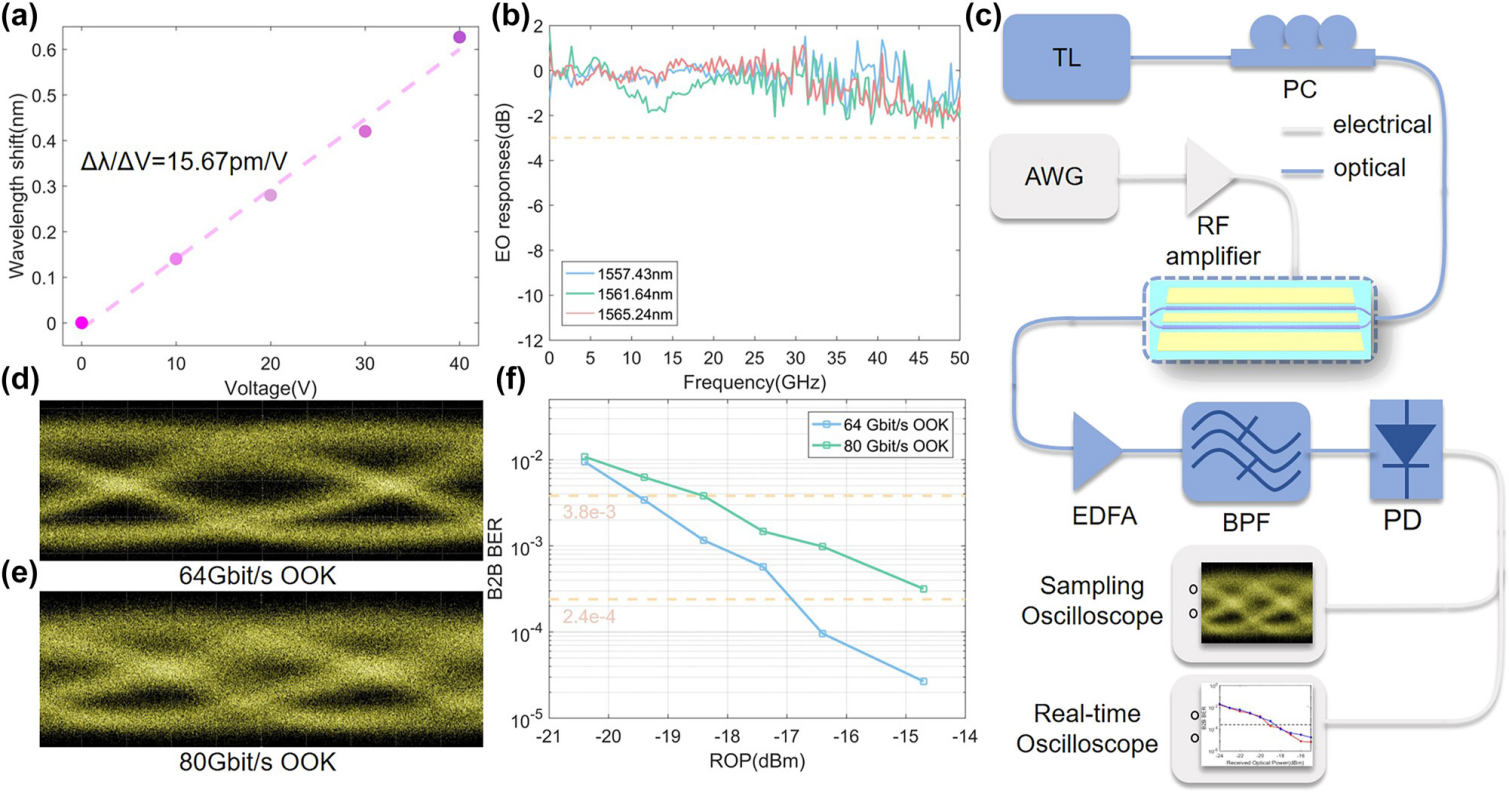
The high-speed measurement. (a) Measured and linear fitted wavelength shift as a function of the applied DC voltage. (b) Measured EO responses for the fabricated SL-MZM under different working wavelengths spanning from 1557.43 nm to 1565.24 nm. (c) Experimental setups for measuring the eye diagram and B2B BER. AWG, arbitrary wave generator; TL, tunable laser; PC, polarization controller; EDFA, erbium-doped fiber amplifier; BPF, bandpass filter; PD, photodetector. Measured optical eye diagrams for SL-MZM at data rates of (d) 64 Gbit/s OOK and (e) 80 Gbit/s OOK. (f) Measured B2B BERs as a function of ROP values for 64 Gbit/s OOK and 80 Gbit/s OOK signals.

To investigate the impact of the SL effect in an EO modulator, the fabricated SL-MZMs with the SL waveguides on both arms shown in Figure 5(b) and (c) were further measured. In order to balance the IL and modulation depth, structural parameters of *W* = 0.4 μm, *δ* = 0.24 μm, *P* = 20, and *N* = 20 were adopted in the fabricated SL-MZM corresponding to a measured optical *n*_g_ of ∼4.0. Figure 7(a) shows the measured direct current (DC) modulation performance of the device. The DC voltage was swept from 0 V to 40 V with step of 10 V and the interference wavelength of the SL-MZM was monitored. The wavelength shifts exhibit a linear curve with an EO tuning efficiency of ∼15.7 pm/V, corresponding to a modulation efficiency *V*_*π*_*L* of ∼1.29 V cm, which is nearly the same as the simulated result shown in Figure 4(c). The high-frequency responses for the EO modulation were also measured and are shown in Figure 7(b). One can find that flat EO responses are presented with modulation bandwidths clearly >50 GHz at different wavelengths. This high EO bandwidth of the present device clearly surpasses those of plasmonic-based MZMs [14] and other SL-MZMs using traditional Bragg grating structures [23]. We further studied the application of the device in high-speed data transmission. The experimental setups for measuring the eye diagram and back-to-back (B2B) bit error rates (BERs) are shown in Figure 7(c). As shown in Figure 7(d) and (e), the transmitted raw eye diagrams were recorded for 64 Gbit/s and 80 Gbit/s on–off keying (OOK) modulation using the present SL-MZM, showing clear open eyes with dynamic ERs of ∼2 dB. Here, the driving microwave signal was amplified to 8.5 V (peak-to-peak) with a high-speed electrical amplifier. We then performed the B2B BER measurement under different received optical power (ROP). The BERs were calculated off-line using linear feed-forward equalization (FFE) and Volterra nonlinear equalization (VNLE)-based DSP algorithms for data rate of 64 Gbit/s and 80 Gbit/s, respectively. The B2B BERs can drop below the KP-4 forward error correction (KP-4 FEC) threshold (2.4 × 10^−4^) at the data rate of 64 Gbit/s OOK modulation, and hard-decision forward error correction (HD FEC) threshold (3.8 × 10^−3^) at the data rate of 80 Gbit/s OOK modulation, respectively.

## 4 Discussion

Table 1 summarizes recent demonstrations of compact modulator structures on TFLN. Apparently, the present SL waveguides using multiple coupled Bragg resonators exhibit a large working wavelength bandwidth, as well as a small insertion loss. The wide working wavelength bandwidth also helps improve the stability of the modulator with respect to the environmental changes, such as temperature, as compared to other demonstrations. Although the achieved optical *n*_g_ is smaller than the Bragg grating structure [23], the good overall performances of the fabricated SL-MZM here allow to achieve the best EO modulation bandwidth, as well as a high-speed transmission for practical data patterns. The IL performance of the present device can be further improved by using an oxide overcladding or an annealing treatment [24] to decrease the propagation loss. Since the modulation performances here are still limited by the insufficient dynamic ER, the improved propagation loss can facilitate to adopt a longer modulation section, e.g., a larger *N*, to achieve a larger modulation depth. In addition, to achieve a better modulation bandwidth, an optimal design of the traveling-wave electrode is necessary, considering the significant index mismatch between the optical wave and the microwave, which dramatically damps the EO responses. Using periodic capacitively loaded traveling-wave (CLTW) electrode [6, 7] and meander-line electrode [7, 25] designs can effectively mitigate the index mismatch, and hence further promote the modulation bandwidth. With the above improvements, the present modulator structure is expected to achieve higher modulation speed.

**Table 1:** Comparisons of reported compact modulators on the TFLN platform.

Platform	Structure	Maximal	IL	Working wavelength	Modulation	*V*_*π*_*L*/tuning	EO	OOK data
		optical *n*_g_		bandwidth	length	efficiency	bandwidth	rate
TFLN [9]	MR resonator	–	1.5 dB	0.03 nm^a^	500 μm	7 pm/V	30 GHz	40 Gbit/s
TFLN [12]	PC cavity	–	2.2 dB	0.01 nm^a^	30 μm	16 pm/V	17.5 GHz	11 Gbit/s
TFLN [11]	FP cavity	–	0.9 dB	0.15 nm^a^	50 μm	7 pm/V	25 GHz	40 Gbit/s
Bulk LN [14]	Plasmonic MZM	–	∼20 dB	∼100 nm	15 μm	0.23 V cm	2.8 GHz^a^	–
Silicon-rich nitride TFLN [23]	Bragg grating SL-MZM	10.28	∼10 dB ^b^	∼2 nm	820 μm	0.67 V cm^b^	10 GHz^b^	60 Gbit/s
TFLN (this work)	Coupled Bragg grating resonators SL-MZM	7.5	2.9 dB	∼8 nm	360 μm	1.29 V cm/15.7 pm/V	>50 GHz	80 Gbit/s

^a^Estimated from the quality factor. ^b^At working wavelength of 1548.3 nm.

## 5 Conclusions

We demonstrated SL waveguides using a coupled Bragg resonator structure on the TFLN platform, and the fabricated SL-MZM also shows the feasibility of the SL effect in a high-speed transmission system. To evaluate the SL effect, a series of SL waveguides were fabricated and the maximal optical *n*_g_ of ∼7.5 with a large optical passband width BW_3dB_ = ∼8 nm can be observed, which is nearly 3.4 times as large as that of regular TFLN rib waveguide. By balancing the optical IL and modulation depth, a compact SL-MZM with modulation length of *L* = ∼370 μm is fabricated, which exhibits flat EO responses with a large EO bandwidth >50 GHz and a large working wavelength range. The high-speed OOK transmissions at data rate of 64 Gbit/s and 80 Gbit/s are shown within KP4 FEC threshold and HD FEC threshold, respectively. To the best of our knowledge, it is the first time to observe SL effect experimentally and build a compact SL-MZM on the monolithic TFLN platform. The proposed structure has the potential for further scalability, enabling high-volume and large-scale integration. It represents a new solution for next-generation high-performance EO modulators on the TFLN platform.
